# Overweight risk in early adolescence according to children’s BMI growth channelling changes in international growth standard/references

**DOI:** 10.1017/S136898002510089X

**Published:** 2025-08-22

**Authors:** Mariane Helen de Oliveira, Joana Araújo, Milton Severo, Kévin Allan Sales Rodrigues, Camila Medeiros da Silva Mazzeti, Natalie Grafft, Wolney Lisboa Conde

**Affiliations:** 1 School of Social Work, Boston College, Chestnut Hill, MA, USA; 2 EPIUnit – Instituto de Saúde Pública da Universidade do Porto, Porto, Portugal; 3 Laboratório para a Investigação Integrativa e Translacional em Saúde Populacional (ITR), Universidade do Porto, Porto, Portugal; 4 Departamento de Ciências da Saúde Pública e Forenses, e Educação Médica, Faculdade de Medicina da Universidade do Porto, Porto, Portugal; 5 Departamento de Ensino Pré-Graduado, Instituto de Ciências Biomédicas Abel Salazar da Universidade do Porto, Porto, Portugal; 6 Department of Statistics, Institute of Mathematics and Statistics, University of São Paulo, São Paulo, Brazil; 7 Chronic Conditions and Diet Observatory (OCCA), Faculty of Pharmaceutical Sciences, Food and Nutrition (FACFAN), Federal University of Mato Grosso do Sul, Campo Grande, Brazil; 8 Department of Nutrition, School of Public Health, University of São Paulo, São Paulo, Brazil

**Keywords:** Child, Adolescent, Growth charts, Growth channelling, Overweight

## Abstract

**Objective:**

To compare the international BMI standard/references of the International Obesity Task Force (IOTF), MULT and the WHO and to analyse the association between changes in BMI growth channelling (BMI-GC) during childhood and the risk of being overweight in early adolescence.

**Design:**

Participant data from the Millennium Cohort Study (MCS), young lives (YL) and Generation XXI (G21) cohorts were obtained at three time points. Lin’s concordance correlation coefficient (CCC) and the weighted Kappa coefficient were used to assess the agreement among the BMI standard/references. The relative risk (RR) of being overweight at 9·5–13·5 years, based on an increase in BMI-GC (amplitude ≥ 0·67) between 3·5–6 years and 6·5–9 years, was calculated, with estimates adjusted for sex, ethnicity and socio-economic status.

**Setting:**

Ethiopia, India, Portugal, Vietnam and United Kingdom.

**Participants:**

Totally, 12 624 participants from the MCS, YL and G21 studies.

**Results:**

The prevalence of overweight across the three ages groups was higher when using the WHO standard/reference (12·8–25·9 %) compared with the MULT (17·1–22·9 %) and IOTF (13·0–19·3 %) references. However, substantial agreement (0·95 < CCC ≤ 0·99) was found among these standard/references. Children who increased their BMI-GC by ≥ 0·67 and < 0·86 were more likely to be overweight at 9·5–13·5 years (MULT-RR = 2·49, 95 % CI: 2·00, 3·09/ WHO-RR = 2·47, 95 % CI: 1·96, 3·12/ IOTF-RR = 2·31, 95 % CI: 1·82, 2·93), compared with those who have stayed in their BMI-GC.

**Conclusions:**

A change in the BMI-GC among normal-weight children during childhood was associated with a significantly higher risk of being overweight at 9·5–13·5 years. These findings suggest that monitoring BMI-GC in children could be a tool to intervene and to prevent overweight in early adolescence.

Overweight is a risk factor for several noncommunicable diseases in adults, such as type 2 diabetes mellitus, high blood pressure, stroke, heart disease, dyslipidaemia and certain types of cancer^(1)^. Longitudinal studies have shown that excessive weight during childhood is a risk factor for adulthood obesity with high correlations with BMI over time^(2,3)^. Children with overweight and obesity are more likely to develop these noncommunicable diseases at a younger age and have a higher risk for premature death and morbidities in adulthood^(4)^. Additionally, children affected by obesity can experience breathing difficulties, increased risk of fractures, hypertension, precocious CVD, insulin resistance and psychological disorders^(4,5)^.

The weight status of children and adolescents is typically assessed using BMI growth charts based on growth standards or references^(6)^. A growth standard represents the growth patterns of a ‘healthy’ population and is intended to serve as a prescriptive model for optimal growth^(6,7)^. In contrast, a growth reference describes how a particular population grows (i.e. not restrictive to ‘healthy’ populations), based on observed data, and is therefore descriptive of existing growth trends^(6–8)^. There is one widely used growth standard and several growth references proposed by different organisations that are used globally^(6,7)^. These include the Centers for Disease Control and Prevention growth reference (2000)^(9)^, the WHO Child Growth Standard (WHO/2006)^(7)^, the WHO growth reference (2007)^(8)^, the International Obesity Task Force (IOTF) reference (2012)^(10,11)^, and the MULT reference (2023/2024)^(12–14)^.

The Centers for Disease Control and Prevention Growth Charts (2000) were developed for children and adolescents from birth to 20 years, using nationally representative data from the US health and nutrition surveys^(9)^. For children up to 36 months, the charts include head circumference-for-age, length-for-age, weight-for-age and weight-for-length^(9)^. For those aged 2–20 years, they include stature-for-age, weight-for-age, BMI-for-age and weight-for-stature^(9)^.

The WHO Child Growth Standard (2006) was developed for children under 5 years old, based on a sample of children with optimal health and nutrition conditions, including exclusive breastfeeding for at least 3–4 months^(7)^. It was constructed using longitudinal and cross-sectional data from six countries: Brazil, the USA, Ghana, Norway, India and Oman^(7)^. This standard includes growth charts for length/height-for-age, weight-for-age, weight-for-length, weight-for-height and BMI-for-age^(7)^. The WHO Growth Reference (2007), for children aged 5–19 years, was constructed with non-obese participants born in the 1950s and 1960s in the USA^(8)^. It includes height-for-age, weight-for-age (up to 10 years) and BMI-for-age^(8)^.

The IOTF (2012) reference is for children aged two to 18 years, constructed from data collected between 1963 and 1993 from six locations: Brazil, Hong Kong, the Netherlands, Singapore, the United Kingdom and the USA^10,11)^. This reference includes BMI-for-age growth charts only^(10,11)^.

The MULT growth reference (2023/2024) is a recently developed international reference, including growth charts for height-for-age and BMI-for-age from birth to 20 years and allometric BMI (ABMI)-for-age from 5 to 20 years^(12–14)^. Unlike previous references based on data from upper-middle and high-income countries, the MULT growth reference (2023/2024) was developed using longitudinal data from multiethnic populations in ten countries across low-, middle-, and high-income settings, including Ethiopia, India, Peru, Vietnam, Brazil, Portugal, England, Scotland, Wales and Northern Ireland^(6,12–14)^. This approach ensures broad representation of ethnic and socio-economic backgrounds, with a focus on more recent cohorts, mostly from the 1990s and 2000s, making it more reflective of today’s global populations^(12–14)^.

Although the MULT growth reference (2023/2024) is relatively new and has limited studies assessing its accuracy, preliminary evidence suggests it may perform better than the WHO (2007) and IOTF (2012) growth references^(8,10,12–17)^. Nevertheless, due to the lack of international consensus on the ideal BMI reference for identifying overweight and obesity in children over five, it remains crucial to conduct parallel analyses using the well-established WHO (2007)^(8)^ and IOTF (2012)^(10)^ growth references for comparison.

While the BMI-for-age growth chart is the main indicator to assess weight status in children and adolescents, growth channelling analysis has emerged as a useful method for tracking developmental growth^(6,18–22)^. Growth channelling refers to the tendency of children’s and adolescents’ growth to remain within a narrow ‘channel’ on growth charts during specific developmental periods^(18,21,22)^. From around 3 years of age until the onset of puberty, children’s growth tends to follow a stable trajectory, staying within a defined channel on the chart that represents their growth percentile^(18–22)^. This stability indicates that significant changes in growth are uncommon unless influenced by health or environmental factors^(18,20–22)^. In longitudinal growth studies, a change greater than 0·25 sd in height per year is rarely observed in children during this period^(18,20–22)^. Deviations from the expected growth channel may be indicative of external influences on growth, such as illness, or other developmental disruptions^(18,20–22)^. While this concept has traditionally been applied to height and skeletal maturation analyses, there is evidence that a similar channelling phenomenon occurs in weight and skinfold thickness, albeit with a higher amplitude value^(18)^.

Despite growing interest in understanding growth patterns, a significant gap persists in the literature regarding the investigation of BMI growth channelling (BMI-GC) and its relationship with long-term health outcomes^(21)^. Therefore, the aims of this study were (1) to use the MULT (2023)^(13)^ as the primary reference and compare it with other international BMI standard/references that utilised data from multiple countries, including the WHO (2006/2007)^(7,8)^ and the IOTF (2012)^(10)^ and (2) to analyse the association between changes in BMI-GC during childhood (ages 3·5–6 to 6·5–9) and the risk of being overweight in early adolescence (ages 9·5–13·5) using these three BMI standard/references.

## Methods

### Study population

Data from the longitudinal studies Millennium Cohort Study (MCS), young lives (YL) and Generation XXI (G21) were selected for this study^(23–26)^. The MCS is an ongoing study of 18 818 infants born between 2000 and 2002 from the United Kingdom (England, Northern Ireland, Scotland and Wales)^(23)^. Baseline data were collected when participants who were 9 months old, with follow-up assessments at 3, 5, 7, 11, 17 and 23 years of age^(23,27,28)^. This study used three follow-ups, when the children were 5, 7, 11 years old^(23,27,28)^. The MCS datasets were accessed through the UK Data Service online platform^(23,27,28)^.

YL is an international study that started in 2002, and it was conducted by a team based at the University of Oxford^(24,25)^. It includes data from 12 000 children across four developing countries (Ethiopia, India, Peru and Vietnam)^(24,25)^. The study is divided into two cohorts: the YL younger cohort with children assessed from one year old and the YL older cohort with children assessed from eight years old forward^(24,25)^. The YL younger cohort participants were re-evaluated at approximately ages 5, 8, 12 and 15, while the YL older cohort participants were re-evaluated around ages 12, 15, 19 and 22^(24,25)^. For this study, we used data only from the YL younger cohort and from the follow-ups when the children were 5, 8 and 12 years old, specifically using data from Ethiopia, India and Vietnam, as these countries provided anthropometric data during the childhood period of interest^(24,25)^. The YL datasets were obtained through the UK Data Service online platform^(24,25,29–31)^.

The G21 is an ongoing study developed by a team of researchers from the University of Porto, conducted in the metropolitan region of the city of Porto, Portugal^(26)^. It is a population-based birth cohort of newborns and their mothers recruited between April 2005 and August 2006^(26)^. The initial sample consisted of 8647 newborns and 8495 mothers^(26,32)^. Children have been re-evaluated at 4, 7, 10 and 13 years of age^(26,32)^. This study used data from three follow-ups, conducted when the children were 4, 7 and 10 years old. The G21 databases were obtained through the authorisation from the *Instituto de Saúde Pública da Universidade do Porto* ^(26,32)^.

The anthropometric (height and weight) and demographic (sex, age and socio-economic) data collection of the MCS, YL and G21 were performed by trained professionals who followed a standardised examination protocol to ensure data quality of the measurements and data^(23–26)^.

### Data processing and study eligibility

**Figure 1.**
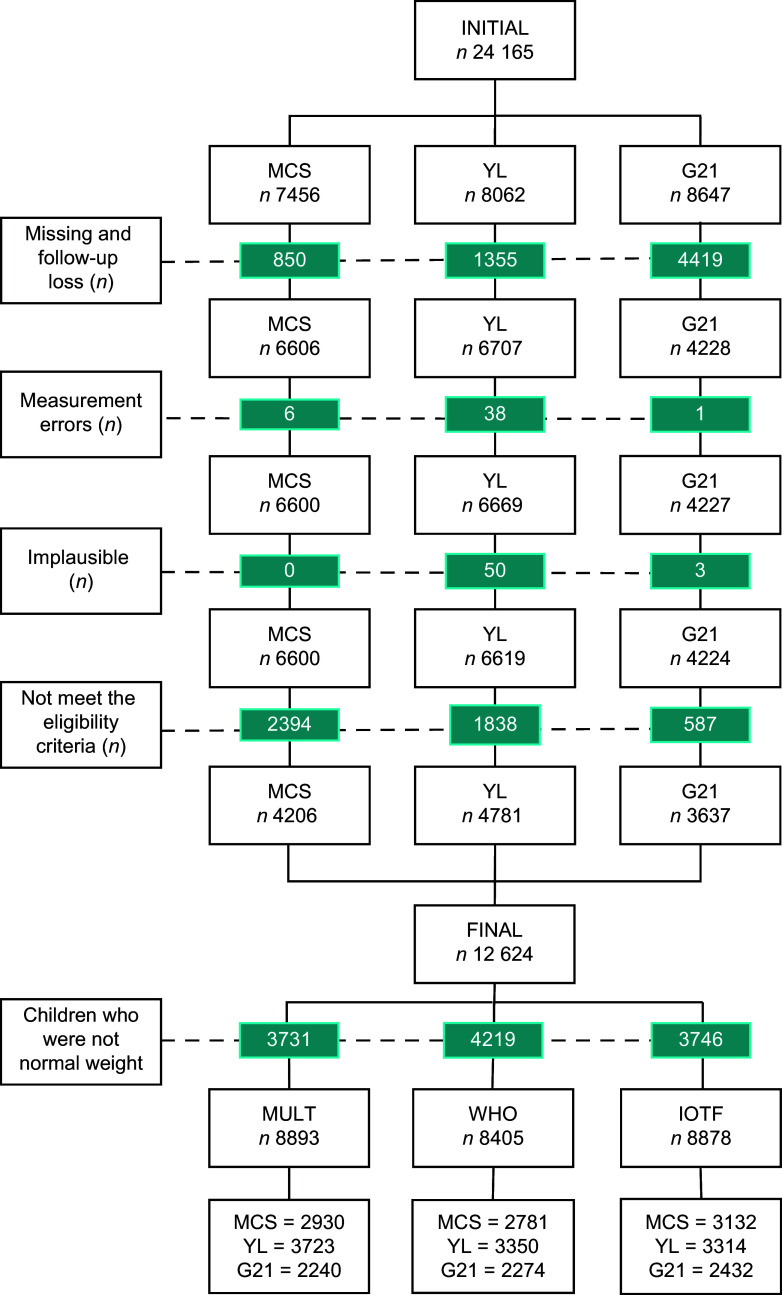
Flowchart of participant selection. Measurement errors: children and adolescents who had decreased their height over the years. Implausible values: height-for-age *z*-score below –6 sd or above +6 sd or BMI-for-age *z*-score below –5 sd or above +5 sd. *n*, number of participants; IOTF, International Obesity Task Force

In the selection process, data of 7456 children from MCS, 8062 children from YL and 8647 from G21 were gathered and the following exclusions were performed: missing anthropometric and/or demographic data or follow-up losses (*n* 6624 participants): measurement error, in which participants decreased their height (≥ 0·5 cm) over the years (*n* 45 participants); implausible values considered as height-for-age *z*-score below –6 sd or above +6 sd or BMI-for-age *z*-score below –5 sd or above +5 sd based on the WHO standard/reference values (*n* 53 participants) and children (*n* 4819 participants) who did not meet the following criteria: (a) evaluated at three specific age periods (3·5–6 years, 6·5–9 years and 9·5–13·5 years); (b) age difference between the second and the first measurement of ≥ 24 months and ≤ 54 months; (c) age difference between the third and the second measurement of ≥ 30 months and ≤ 60 months; d) ethnicity classification into one of four the categories: White, Asian Indians, Black or East and Southeast Asians and e) sample size per country ≥ 300 participants^(7,8,33)^. The final analytic sample was 12 624 (4206 from MCS, 4781 from YL and 3637 from G21), as shown in Figure 1.

Regarding the demographic variables, sex was categorised as male or female according to the designation recorded at birth. Socio-economic status was classified into five country-specific wealth quintiles (1st being the poorest and 5th the wealthiest quintile), based on study-specific socio-economic data collected. In the YL, the socio-economic status was determined using the wealth index variable, which is an average of the housing quality, consumer durables and services indexes^(24,25)^. In the MCS and G21 studies, the income variable was used; however, the G21 considered only family income, while the MCS index was calculated using both household income and family consumption/expenditure^(23,26)^.

### Statistical analyses

The prevalence of the weight status of the sample was calculated according to the BMI-for-age standard/references of IOTF (2012)^(10)^, MULT (2023)^(13)^ and WHO (2006/2007)^(7,8)^. The children’s *z*-score of BMI-for-age in their three evaluations (3·5–6 years; 6·5–9 years and 9·5–13·5 years) was calculated for each BMI standard/reference and classified into one category: underweight, normal weight, overweight and obesity^(7,8,10,13)^. For the MULT BMI reference^(13)^, the following cut-off points were chosen: underweight < 3rd percentile; overweight ≥ 85th percentile and obesity ≥ 98th percentile. The obesity cut-off point was selected as suggested in a previous study, and the underweight and overweight cut-offs are the same applied by the WHO (2006/2007)^(7,8,16)^. For each BMI growth standard/references, the BMI-GC was calculated according to the BMI *z*-score difference (∆1) between the second and first evaluations (6·5–9 years; 3·5–6 years) and classified into three categories of amplitudes: < 0·67 (reference category), ≥ 0·67 to < 0·86 and ≥ 0·86. The reference category had an amplitude of < 0·67 since researchers pointed out that 0·5 is usually the good range for growth channelling in height, while for weight and skinfolds the amplitude should be higher, and a study that analysed growth channelling in children applied the value of 0·67 sd as a cut-off^(18,34)^.

For the BMI standard/reference comparisons, Lin’s concordance correlation coefficient (CCC) was applied to verify the agreement among the BMI *z*-scores, and the weighted Kappa was applied to analyse the reliability among the weight status classified by the IOTF (2012), MULT (2023) and WHO (2006/2007) BMI standard/references^(7,8,10,13,35,36)^. The CCC measures the concordance agreement on a continuous measure obtained by two methods, and it combines measures of both precision and accuracy to determine how far the observed data deviate from the line of perfect concordance, while the weighted Kappa evaluates the level of the reliability between two methods for ordinal categorical outcomes^(35,36)^. The CCC and Kappa were applied to the entire sample and separated by sex and age groups (3·5–6 years; 6·5–9 years and 9·5–13·5 years).

For the overweight risk analysis, only children who were normal weight at the two first evaluations according to the BMI standard/references were included: IOTF/2012^(10)^ (8878 participants), MULT/2023^(13)^ (8893 participants) and WHO/2006–2007^(7,8)^ (8405 participants). This classification resulted in distinct participant counts for each standard/reference due to the varying criteria and cut-off points used to determine normal weight. The relative risk (RR) of being overweight at 9·5–13·5 years, according to the increase in BMI-GC (amplitude ≥ 0·67) between ages of 3·5–6 years and 6·5–9 years was calculated, and estimates were adjusted for sex, ethnicity and socio-economic status^(37)^.

For the RR analysis, the log-binomial regression with constrained optimisation was performed using the *lbreg* package in R^(37,38)^. This package was selected since traditional algorithms for calculating maximum likelihood estimation (MLE) were designed for the scenario in which the MLE is inside the parametric space so that the convergence of these algorithms is impaired when the MLE is close to the boundary of the parametric space^(37,38)^. On the other hand, *lbreg* package provides the MLE of log-binomial regression when the MLE is on the border of the parametric space, and it also provides point and interval estimates of RR^(37,38)^. In addition, the quality of the model fit was assessed using the Hosmer and Lemeshow test (1980)^(39)^. All statistical analyses were performed using R software, version 4.2.3 for Windows^(38)^.

## Results

**Figure 2.**
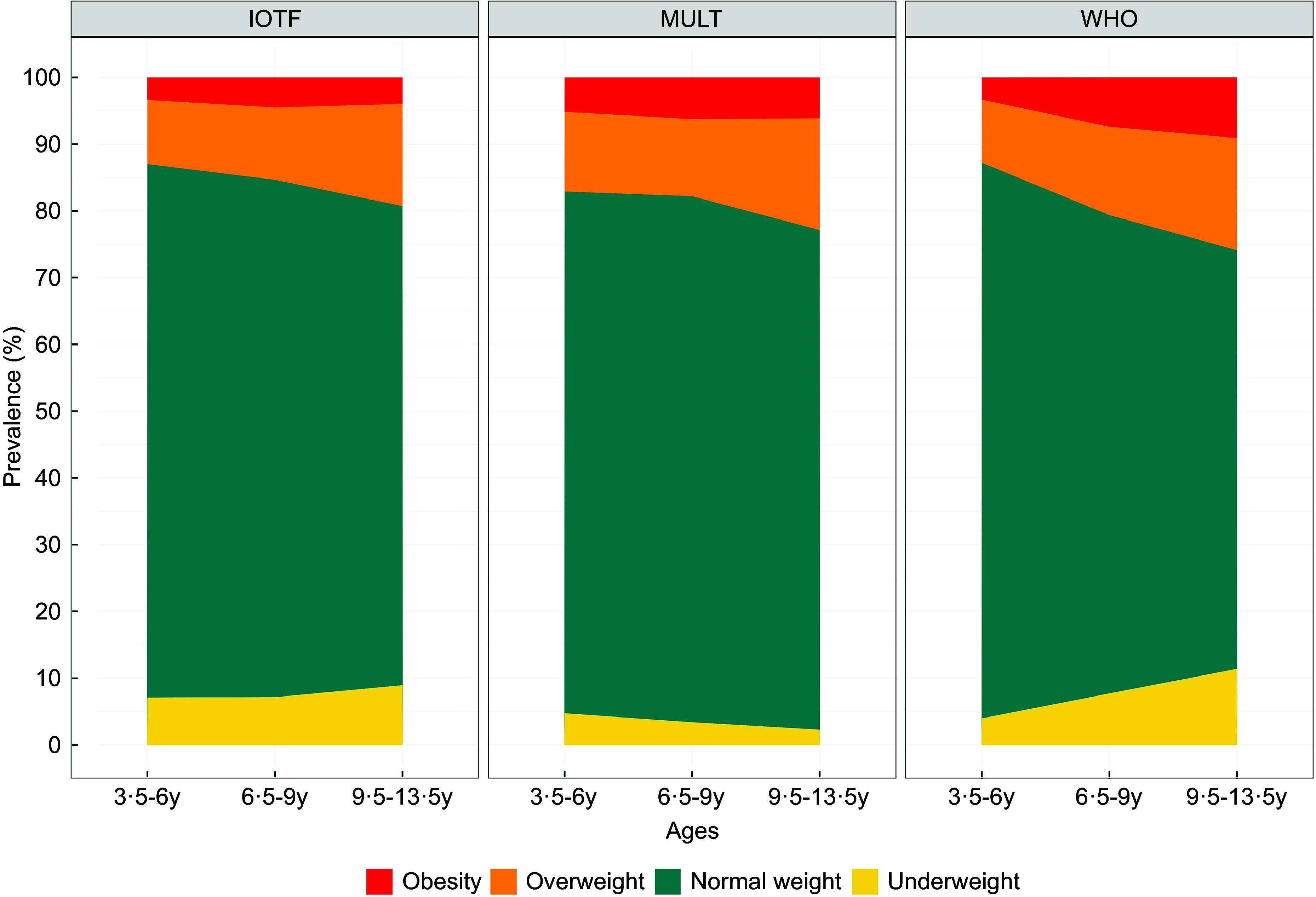
Prevalence of underweight, normal weight, overweight and obesity estimated by the MULT, WHO and IOTF, standard/references, in three periods of time. IOTF, International Obesity Task Force

After the inclusion and exclusion criteria, 12 624 children (51·4 % males) were included in the growth standard/reference comparison analysis. The majority of the sample was composed of White children (61·8 %), and from the 3rd wealth quintile (26·1 %), as shown in Table 1. The weight status differs across the different international BMI standard/references, as shown in Figure 2, with additional details provided in online supplementary material, Supplemental Table 1. For the underweight category, the prevalence at different ages ranged from 4·7 to 2·3 % when applying the MULT reference, while estimates ranged from 4·0 to 11·4 % with the WHO standard/reference, and from 7·1 to 9·0 % with the IOTF reference.

**Table 1.** Characteristics of the study population

Entire sample (*n* 12 624)		
	*n*	%
Sex		
Male	6492	51·4 %
Female	6132	48·6 %
Race/Ethnicity		
White	7804	61·8 %
Black	1471	11·7 %
Asian Indian	1678	13·3 %
East and Southeast Asians	1671	13·2 %
Socio-economic status		
First quintile	1623	12·9 %
Second quintile	2673	21·2 %
Third quintile	3295	26·1 %
Fourth quintile	3184	25·2 %
Fifth quintile	1849	14·6 %

*n*, number of participants.

Applying Lin’s CCC (Table 2) substantial agreement (CCC > 0·95 and ≤ 0·99) was found among the MULT, WHO and IOTF, BMI standard/references for the entire sample and for the majority of the age groups, with the exception of the analysis between MULT and IOTF for the age group of 3·5–6 years and WHO and IOTF for the age group of 9·5–13·5 years that presented an almost perfect agreement (CCC > 0·99). When the analysis was stratified by sex, the agreement between WHO and IOTF standard/references was higher among females (CCC > 0·99) than in males for all age groups. The lower performance, presenting moderate agreement, was between MULT and the WHO MULT BMI references for males aged 9·5–13 years old (CCC = 0·922).

**Table 2.** BMI-for-age *z*-score concordance (CCC) and weight status agreement (Kappa) among MULT, WHO and IOTF BMI standard/references, by age group and sex

	Entire sample 3·5 ≤ 13·5 y (Mean 7·9 y; sd 2·7 y)					3·5–6 y (Mean 4·9 y; sd 0·5 y)					6·5–9 y (Mean 7·5 y; sd 0·5 y)				9·5–13·5 y (Mean 11·2 y; sd 0·9 y)			
	*n*	CCC	95 % CI	Kappa	95 % CI	*n*	CCC	95 % CI	Kappa	95 % CI	CCC	95 % CI	Kappa	95 % CI	CCC	95 % CI	Kappa	95 % CI
MULT x WHO																		
Overall	37 872	0·967	0·966, 0·967	0·784	0·778, 0·790	12 624	0·989	0·989, 0·989	0·728	0·715, 0·741	0·979	0·979, 0·980	0·847	0·839, 0·857	0·938	0·937, 0·939	0·764	0·755, 0·774
Male	19 476	0·957	0·956, 0·957	0·756	0·748, 0·765	6492	0·983	0·983, 0·984	0·730	0·711, 0·748	0·972	0·972, 0·973	0·802	0·788, 0·816	0·922	0·920, 0·924	0·733	0·719, 0·746
Female	18 396	0·978	0·978, 0·979	0·813	0·805, 0·821	6132	0·995	0·995, 0·995	0·726	0·708, 0·745	0·987	0·987, 0·988	0·895	0·884, 0·901	0·956	0·955, 0·957	0·799	0·786, 0·812
MULT x IOTF																		
Overall	37 872	0·986	0·985, 0·986	0·805	0·747, 0·762	12 624	0·993	0·993, 0·993	0·816	0·805, 0·826	0·988	0·988, 0·989	0·834	0·824, 0·844	0·976	0·975, 0·977	0·769	0·759, 0·780
Male	19 476	0·985	0·985, 0·985	0·771	0·762, 0·780	6492	0·995	0·995, 0·995	0·822	0·807, 0·838	0·989	0·989, 0·990	0·786	0·770, 0·802	0·971	0·970, 0·972	0·715	0·699, 0·731
Female	18 396	0·986	0·986, 0·987	0·837	0·829, 0·845	6132	0·991	0·991, 0·991	0·809	0·794, 0·824	0·988	0·987, 0988	0·878	0·866, 0·889	0·981	0·980, 0·982	0·823	0·809, 0·836
WHO x IOTF																		
Overall	37 872	0·990	0·989, 0·990	0·775	0·769, 0·781	12 624	0·989	0·988, 0·989	0·691	0·677, 0·701	0·993	0·993, 0·993	0·819	0·809, 0·828	0·988	0·987, 0·988	0·784	0·775, 0·793
Male	19 476	0·985	0·985, 0·985	0·723	0·714, 0·732	6492	0·983	0·982, 0·984	0·686	0·665, 0·701	0·989	0·989, 0·989	0·751	0·736, 0·766	0·984	0·983, 0·984	0·718	0·704, 0·731
Female	18 396	0·995	0·995, 0·995	0·828	0·821, 0·836	6132	0·995	0·995, 0·995	0·698	0·678, 0·718	0·998	0·997, 0·998	0·887	0·876, 0·897	0·992	0·992, 0·992	0·855	0·844, 0·866

Mean, average age in years; y, years; *n*, number of participants; CCC, Lin’s concordance correlation coefficient; Kappa, weighted Kappa coefficient; IOTF, International Obesity Task Force.

In the analysis of the weight status, applying weighted Kappa coefficient (Table 2) there was a substantial reliability (0·60 < Kappa ≤ 0·80) between WHO and IOTF BMI standard/references for males in all age groups. On the other hand, there was an almost perfect reliability (Kappa > 0·80) between MULT and IOTF in all age groups for females. Although the reliability between WHO and IOTF was substantial (0·60 < Kappa ≤ 0·80) for most of the age groups, Kappa values lower than 0·60 were not found, which indicated good reliability among the BMI standard/references.

For all the BMI references, there was a high risk of overweight in early adolescence for participants who were normal weight in the first two evaluations and who changed their BMI-GC ≥ 0·67 and < 0·86 (MULT RR = 2·49, 95 % CI: 2·00, 3·09/WHO RR = 2·47, 95 % CI: 1·96, 3·12/IOTF RR = 2·31, 95 % CI: 1·82, 2·93) in comparison with children who stayed in the growth channelling of ≥ 0 and < 0·67, as shown in Table 3. The risk was even higher for children who increase their BMI-GC ≥ 0·86 (MULT RR = 3·30, 95 % CI: 2·80, 3·89/WHO RR = 3·28, 95 % CI: 2·78, 3·88/IOTF RR = 3·56, 95 % CI: 3·03, 4·18).

**Table 3.** Risk of overweight in early adolescence (9·5–13·5 years) according to the BMI-GC changes during childhood. Log-binomial regression adjusted for sex, ethnicity and socio-economic status

	MULT (*n* 8893)			WHO (*n* 8405)			IOTF (*n* 8878)		
	RR	CI (95 %)	*P* value	RR	CI (95 %)	*P*-value	RR	CI (95 %)	*P* value
(Intercept)	0·11	0·09, 0·13	< 0·001	0·14	0·12, 0·17	< 0·001	0·08	0·06, 0·09	< 0·001
Growth channelling									
< 0·67	1	–	–	1	–	–	1	–	–
≥ 0·67 to < 0·86	2·49	2·00, 3·09	< 0·001	2·47	1·96, 3·12	< 0·001	2·31	1·82, 2·93	< 0·001
≥ 0·86	3·30	2·80, 3·89	< 0·001	3·28	2·78, 3·88	< 0·001	3·56	3·03, 4·18	< 0·001
Sex									
Male	1	–	–	1	–	–	1	–	–
Female	0·93	0·81, 1·05	0·236	0·94	0·84, 1·05	0·265	1·03	0·90, 1·18	0·624
Race/ethnicity									
White	1	–	–	1	–	–	1	–	–
Asian Indian	0·34	0·26, 0·45	< 0·001	0·38	0·30, 0·49	< 0·001	0·39	0·29, 0·52	< 0·001
Black	0·05	0·02, 0·10	< 0·001	0·04	0·02, 0·08	< 0·001	0·04	0·02, 0·11	< 0·001
East and Southeast Asians	0·40	0·31, 0·51	< 0·001	0·37	0·29, 047	< 0·001	0·31	0·23, 0·42	< 0·001
Socio-economic									
Fifth wealth quintile	1	–	–	1	–	–	1	–	–
Fourth wealth quintile	1·09	0·89, 1·34	0·376	1·03	0·85, 1·23	0·783	1·21	0·97, 1·52	0·497
Third wealth quintile	1·03	0·84, 1·27	0·806	0·97	0·81, 1·17	0·777	1·20	0·96, 1·50	0·115
Second wealth quintile	1·00	0·81, 1·24	0·921	1·01	0·83, 1·22	0·954	1·08	0·86, 1·37	0·092
First wealth quintile	1·24	1·00, 1·53	0·045	1·26	1·05, 1·53	0·015	1·44	1·14, 1·81	0·002

RR, relative risk; *n*, number of participants, IOTF, International Obesity Task Force.

Reference category: 1.

Regarding ethnicity and socio-economic status, for all BMI standard/references, Black, Asian Indian and East and Southeast Asians presented a lower risk of being affected by overweight than White children, and those from the 1st wealth quintile presented a higher risk in comparison to the children from the fifth wealth quintile. Even though the sex variable was included in the model, no differences were found across the BMI standard/references. Lastly, the Hosmer and Lemeshow test showed a *P* value >0·05 for the model using the IOTF reference (MULT = 0·03; WHO = 0·01; IOTF = 0·15), which indicates a good fit of this model.

## Discussion

In this study, the weight status estimated differed significantly across the three growth standard/references. The WHO BMI growth standard/reference (2006/2007)^(7,8)^ generally classified more children and adolescents as underweight, overweight and obese than MULT (2023)^(13)^ and IOTF (2012)^(10)^. These findings are similar to several studies that reported that the WHO BMI growth standard/reference (2006/2007)^(7,8)^ usually estimates a higher prevalence of overweight and obesity than the IOTF (2012) BMI reference^(10,40–42)^. Otherwise, for underweight classification, the BMI reference from MULT (2023)^(13)^ presented opposite results than the ones found using the BMI standard/references from WHO (2006/2007)^(7,8)^ and IOTF (2012)^(10)^, since its prevalence estimate decreased over the years instead of increasing. For overweight and obesity, the MULT (2023)^(13)^ reference presented lower estimates than the WHO (2006/2007)^(7,8)^ but higher estimates than IOTF (2012)^(10)^ appearing to be a middle ground between WHO (2006/2007)^(7,8)^ and IOTF (2012)^(10)^ BMI standard/references. These findings indicate that the choice of the BMI standard/reference influences the prevalence of overweight/obesity in children and that these differences are generated by the growth reference study design, population and criteria to establish their cut-off points^(6,42)^.

Moreover, the overestimates in overweight and obesity prevalence applying the 2007 WHO growth reference^(8)^ occurred only from the second assessment forward, when the participants were older than 5 years old. These findings support the results of several studies that pointed out that the 2006 WHO child growth standard^(7)^ seems to be adequate to be used internationally since it was constructed with recent data of multiethnic children who had environmental and health conditions adequate for optimal development, while for children from 5 years old, the 2007 WHO growth reference^(8)^ seems to be inaccurate for diagnosing overweight and/or obesity^(6)^. A systematic review^(6)^ pointed out that for some European countries such as Slovakia, Portugal, Italy and Poland, the IOTF (2012)^(10)^ presented a better performance than the WHO (2006/2007)^(7,8)^ BMI standard/reference, while for Asian countries China, Iran and Saudi Arabia there are recommendations for the use of national BMI growth charts instead.

Additionally, a cross-sectional study^(16)^ that assessed obesity diagnosis through the body composition analysis in US children and adolescents from 8 years old pointed out that among the BMI references – MULT (2023)^(13)^, WHO (2007)^(8)^ and IOTF (2012)^(10)^ – the WHO (2007)^(8)^ reference had the lowest performance, suggesting the use of either MULT (2023)^(13)^ or IOTF (2012)^(10)^ BMI references for the US population. In this study, the female’ weight status classified by the BMI references showed that the MULT (2023)^(13)^ and IOTF (2012)^(10)^ references presented more agreement among them than with the WHO (2006/2007)^(7,8)^ growth standard/reference. This can be explained by the higher ethnic sample diversity of MULT (2023)^(13)^ and IOTF (2012)^(10,11)^ references, in comparison with the 2007 WHO reference^(8)^, even though there is an absence of African countries in the sample of the IOTF (2012)^(10,11)^.

Despite the international growth references differences, this study highlights the importance of monitoring the BMI-GC changes during childhood. Notable, an increase higher than 0·67 sd in the BMI-GC more than doubles the risk of being affected by overweight in early adolescence, regarding the BMI reference applied. This finding emphasises the need for ongoing surveillance of BMI changes as children grow. However, research on BMI-GC is still scarce, even though studies pointed out the association between the increase in BMI *z*-score and the complications of overweight in normal-weight children, and the early increase in weight *z*-score and adiposity gain in toddlers^(34,43)^.

Lower socio-economic status was associated with a higher risk to developed overweight, consistent with studies showing that children from lower-income families presented a higher risk for obesity. This risk is especially evident among those living in unsafe neighbourhoods, spending more time indoors, watching more television and not having healthy eating habits^(44,45)^. Additionally, although overweight prevalence affects all countries, which makes it a public health issue, the rising trends in children’s and adolescents’ BMI stagnated in high-income countries, while it is still increasing in lower-income countries, especially those from Asia^(4,46)^.

Moreover, overweight during childhood and adolescence can influence linear growth and, in some cases, result in a slightly shorter final height^(47)^. Children affected by obesity often experience accelerated growth in the early stages of life due to the stimulation of growth hormones associated with excess body fat^(47)^. While this initial growth spurt may seem advantageous, it can lead to early bone maturation and premature closure of growth plates, thereby limiting further longitudinal growth^(47)^. Hormonal changes due to obesity, such as elevated insulin and sex hormone levels, can also disrupt growth patterns^(47)^. This combination of early bone maturation and hormonal dysregulation may partly explain the tendency for lower final height observed in children and adolescents affected by obesity^(47)^.

There is a need for monitoring children’s weight status for preventing or managing overweight and obesity conditions^(4,6,40)^. In 2015, the Canadian Task Force on Preventive Health Care published recommendations for the prevention and management of overweight and obesity in children and youth in primary care, which included the monitoring of changes in BMI *z*-score^(48)^. In this way, the BMI-GC can be a monitoring tool, especially because an increase in it is positively associated to overweight, indicating a need for an intervention, even for those who presented normal weight previously.

Regarding the strengths of this study, it is the first one that compared the MULT (2023)^(13)^ BMI reference to WHO (2006/2007)^(7,8)^ and the IOTF (2012)^(10)^ BMI standard/references, using standardised longitudinal surveys, which provided an analysis of the BMI over time. Moreover, this study included data from multiple longitudinal surveys conducted in various countries, allowing for the assessment of BMI trends in a multiethnic population. The anthropometric data were collected by trained professionals, which is supposed to reduce the odds of measurement errors and social desirability bias^(49)^. Additionally, to ensure data quality and model reliability, the exclusion of measurement errors and implausible values was performed. The log-binomial regression with constrained optimisation was applied in the analyses of the BMI-GC, which provided the ratio of the risks, which is an association measure well known to be used in epidemiological studies with longitudinal data^(37)^.

Nevertheless, there are certain limitations in our study. One limitation is the absence of information regarding body composition or health outcomes that could be used to indicate which BMI standard/references presented the best performance to screen overweight and obesity. Additionally, examining growth over a relatively short period during adolescence and defining growth channels based on only two measurement points may restrict our understanding of the long-term patterns and implications of BMI-GC. Another limitation is the use of city data instead of a national sample in the G21 study, even though it is a population-based study from the city of Porto, which presented similar patterns to the Portuguese data^(50)^. Furthermore, individuals with missing data or those lost to follow-up were excluded from the analysis, which may have led to selection bias. To ensure robust sample sizes for each country, data from countries with fewer than 300 participants were also excluded. While these exclusions helped maintain the quality and reliability of the analysis, they may have limited the generalisability of the findings to broader populations.

In summary, this study underscores the significance of not only focusing on the weight status cut-off points of BMI growth charts but also monitoring growth channelling. Tracking changes in BMI-GC can provide critical insights into children’s growth patterns. By identifying deviations from expected growth trajectories, targeted interventions and preventive strategies can be implemented to prevent obesity, helping to mitigate the long-term health consequences associated with obesity and related conditions.

## Supporting information

de Oliveira et al. supplementary material de Oliveira et al. supplementary material

## Supplementary material

For supplementary material accompanying this paper visit https://doi.org/10.1017/S136898002510089X
