# Supplementary material for: Overweight risk in early adolescence according to children’s BMI growth channelling changes in international growth standard/references
Source: Public Health Nutr. 2025 Aug 22;28(1):e142. doi: 10.1017/S136898002510089X (PMC12516609; doi:10.1017/S136898002510089X)
Supplement: de Oliveira et al. supplementary material [file S136898002510089Xsup001.docx]

**Table S1.** Weight Status classified by MULT, WHO, and IOTF BMI standard/references across three time periods (3.5-6y, 6.5-9y, 9.5-13y).

|  | MULT | | | WHO | | | IOTF | | |
| --- | --- | --- | --- | --- | --- | --- | --- | --- | --- |
| **3.5-6y** | *n* | % | 95% CI | *n* | % | 95% CI | *n* | % | 95% CI |
| Unwerweight | 882 | 7.0 | 6.5-7.4 | 503 | 4.0 | 3.6-4.3 | 899 | 7.1 | 6.7-7.6 |
| Normal weight | 9 731 | 77.1 | 76.4-77.8 | 10 507 | 83.2 | 82.6-83.9 | 10 085 | 79.9 | 79.2-80.6 |
| Overweight | 1 390 | 11.0 | 10.5-11.6 | 1 194 | 9.5 | 8.9-10.0 | 1 214 | 9.6 | 9.1-10.1 |
| Obesity | 621 | 4.9 | 4.5-5.3 | 420 | 3.3 |  | 426 | 3.4 | 3.1-3.7 |
|  |  |  |  |  |  |  |  |  |  |
| **6.5-9y** |  |  |  |  |  |  |  |  |  |
| Unwerweight | 706 | 5.6 | 5.2-6.0 | 974 | 7.7 | 7.2-8.2 | 904 | 7.2 | 6.7-7.6 |
| Normal weight | 9 800 | 77.6 | 76.9-78.4 | 9 044 | 71.6 | 70.9-72.4 | 9 783 | 77.5 | 76.8-78.2 |
| Overweight | 1 367 | 10.8 | 10.3-11.4 | 1 671 | 13.2 | 12.6-13.8 | 1 369 | 10.8 | 10.3-11.4 |
| Obesity | 751 | 5.9 | 5.5-6.4 | 935 | 7.4 | 6.9-7.9 | 568 | 4.5 | 4.1-4.9 |
|  |  |  |  |  |  |  |  |  |  |
| **9.5-13y** |  |  |  |  |  |  |  |  |  |
| Unwerweight | 518 | 4.1 | 3.8-4.4 | 1 439 | 11.4 | 10.8-12.0 | 1 130 | 9.0 | 8.5-9.4 |
| Normal weight | 9 363 | 74.2 | 73.4-74.9 | 7 916 | 62.7 | 61.9-63.5 | 9 060 | 71.8 | 71.0-72.6 |
| Overweight | 2 020 | 16.0 | 15.4-16.6 | 2 118 | 16.8 | 16.1-17.4 | 1 933 | 15.3 | 14.7-15.9 |
| Obesity | 723 | 5.7 | 5.3-6.1 | 1 151 | 9.1 | 8.6-9.6 | 501 | 4.0 | 3.6-4.3 |

*n*: Number of participants

y: Years

95% CI: 95% confidence interval

WHO: World Health Organization

IOTF: International Obesity Task Force
